# MSDRP: a deep learning model based on multisource data for predicting drug response

**DOI:** 10.1093/bioinformatics/btad514

**Published:** 2023-08-22

**Authors:** Haochen Zhao, Xiaoyu Zhang, Qichang Zhao, Yaohang Li, Jianxin Wang

**Affiliations:** Hunan Provincial Key Lab on Bioinformatics, School of Computer Science and Engineering, Central South University, Changsha 410083, China; School of Computer Science and Engineering, Central South University, Changsha 410083, China; Hunan Provincial Key Lab on Bioinformatics, School of Computer Science and Engineering, Central South University, Changsha 410083, China; School of Computer Science and Engineering, Central South University, Changsha 410083, China; Hunan Provincial Key Lab on Bioinformatics, School of Computer Science and Engineering, Central South University, Changsha 410083, China; School of Computer Science and Engineering, Central South University, Changsha 410083, China; Department of Computer Science, Old Dominion University, Norfolk, VA 23529-0001, United States; Hunan Provincial Key Lab on Bioinformatics, School of Computer Science and Engineering, Central South University, Changsha 410083, China; School of Computer Science and Engineering, Central South University, Changsha 410083, China

## Abstract

**Motivation:**

Cancer heterogeneity drastically affects cancer therapeutic outcomes. Predicting drug response *in vitro* is expected to help formulate personalized therapy regimens. In recent years, several computational models based on machine learning and deep learning have been proposed to predict drug response *in vitro*. However, most of these methods capture drug features based on a single drug description (e.g. drug structure), without considering the relationships between drugs and biological entities (e.g. target, diseases, and side effects). Moreover, most of these methods collect features separately for drugs and cell lines but fail to consider the pairwise interactions between drugs and cell lines.

**Results:**

In this paper, we propose a deep learning framework, named MSDRP for drug response prediction. MSDRP uses an interaction module to capture interactions between drugs and cell lines, and integrates multiple associations/interactions between drugs and biological entities through similarity network fusion algorithms, outperforming some state-of-the-art models in all performance measures for all experiments. The experimental results of *de novo* test and independent test demonstrate the excellent performance of our model for new drugs. Furthermore, several case studies illustrate the rationality for using feature vectors derived from drug similarity matrices from multisource data to represent drugs and the interpretability of our model.

**Availability and implementation:**

The codes of MSDRP are available at https://github.com/xyzhang-10/MSDRP.

## 1 Introduction

In recent years, cancer becomes one of the leading causes of death, seriously threatening human health. Cancer heterogeneity leads to differences in tumor growth rate, invasion ability, drug sensitivity, and prognosis, which greatly limits many therapeutic strategies (Fan *et al.* 2019). Therefore, being able to predict drug response *in vitro* is expected to help physicians target specific therapies for different types of tumors while minimizing drug toxicity for patients and saving medical costs (Adam *et al.* 2020). Recently, high-throughput screening techniques pave the way for researchers to analyze genomic patterns of cancer and measure drug candidate sensitivity *in vitro*. Therefore, discovering anticancer drug response based on the patient’s clinical molecular features accurately and robustly becomes a significant challenge in the era of precision medicine. In recent years, the development of high-throughput screening techniques facilitates the initiation of several large cancer genome projects to analyze the genomic patterns of cancer. For example, Cancer Cell Line Encyclopedia (CCLE) (Barretina *et al.* 2012) performs large-scale deep sequencing of more than 1000 human cancer cell lines covering more than 30 tissue sources, integrating genetic information such as DNA mutation, gene expression, and chromosome copy number. Genomics of Cancer Drug Sensitivity (GDSC) (Yang *et al.* 2013) is the most frequently used dataset in current drug response prediction studies, which provides researchers with multiomics data including genome, transcriptome, proteome, and methylome data.

Openness and availability of large-scale datasets related to drugs and cell lines facilitate development of drug response prediction methods (Liu *et al.* 2019, 2020, Hostallero *et al.* 2022, Nguyen *et al.* 2022). For example, Peng *et al.* (2022) proposed a method using parallel heterogeneous graph convolutional networks to predict drug response. The method linearly transforms the gene expression of cell lines and molecular fingerprints of drugs into vector space of the same dimension and inputs them into the interaction model consisting of a parallel graph convolutional network layer and a neighborhood interaction layer. Chawla *et al.* (2022) presented a predictive approach for inferring drug response in cancers using gene expression data. The method demonstrates the benefits of considering pathway activity estimates in tandem with drug descriptors as features. Zhu *et al.* (2022) proposed a deep learning method with similarity enhancement. The method constructs twin graph neural networks for drug response prediction (TGDRP) and a similarity augmentation (SA) module to fuse fine-grained and coarse-grained information of drugs and cell lines. More recently, Wang *et al.* (2023) predicted drug–cell line pair (DCP) drug response by constructing a sparse network with DCP similarity information.

Although some methods use SMILES sequences or similarities of drugs to predict drug response, most of them obtained drug features based on a single drug description (drug structures), without considering the relationships between drugs and biological entities (e.g. targets, diseases, and side effects). Some studies show that the associations/interactions between drugs and biological entities are crucial to the recognition of drug functions (Chen *et al.* 2021). Considering the above limitations, we propose a deep learning framework, named MSDRP for drug response prediction. MSDRP uses a similarity network fusion (SNF) (Wang *et al.* 2014) algorithm and an interaction module to integrate multiple heterogeneous data sources. More specifically, we first collect the multisource data for drugs and cell lines, and calculate multiple similarity matrices for drugs and multiple feature matrices for cell lines. Then we use the SNF algorithm to fuse the similarity matrices calculated based on the drug structure and fill the similarity matrices calculated based on the drug-related biological entities. Moreover, to capture the pairwise correlations between drugs and cell lines, we design an interaction module consisting of an outer product unit and an inner product unit. We concatenate drug embeddings, cell line embeddings, and higher-order correlation embeddings, and then feed them into a prediction module to predict potential drug responses. Experimental results show that our method outperforms other state-of-the-art methods in the drug response prediction. We also conduct *de novo* test and independent test to demonstrate the prediction capability of our model on new drugs. Moreover, we perform a set of ablation experiments to illustrate the effectiveness of each component and the effectiveness of our model. Finally, we conduct several case studies to evaluate the interpretability of our model and the rationality for using feature vectors derived from drug similarity matrices from multisource data. All results show that MSDRP can be used as a powerful tool for drug response prediction.

## 2 Materials and methods

### 2.1 Datasets

In the paper, we formulate drug response prediction task as a regression problem, where IC50 values are served as continuous target values. The benchmark dataset for drug response prediction is obtained from Zhu *et al.*’s (2022) study. The total number of IC50 values in our benchmark dataset is 82 833, which comprises 170 drugs and 580 cell lines. In addition, to discover the sensitivity of new drugs (all related-cell lines are unknown) to cell lines, we collect an independent dataset to test the models. Firstly, we collect the IC50 values of drug–cell line pairs from Peng *et al.* (2022), which contains 436 cancer cell lines and 24 drugs from CCLE database. Then, we remove the duplicated drugs and cell lines in our benchmark datasets. Finally, there are 763 IC50 values in the independent test set, including 12 drugs and 138 cell lines.

### 2.2 Cell features

Let $S_{\text{cell}}=\left\{c_{1},c_{2},\ldots c_{m}\right\}$ represent the set of all *m* different cell lines. We adopt the collection approach similar to Zhu *et al.*’s (2022) study and obtain multiomics data of cell lines in CCLE database. We construct four feature matrices of cell lines, including three omics matrices based on the multiomics data and a similarity matrix calculated by Chebyshev distance based on the known IC50 values between drugs and cell lines. In feature matrix, a row indicates the feature vector for a cell line. More specifically, we first collect three types of omics data for the cell lines in the benchmark dataset, including gene expression, somatic mutation, and copy number variation. Then, we obtain 706 cancer-related genes from COSMIC database (Tate *et al.* 2019). Finally, we construct three omics matrices $\left\{M_{\;\text{exp}\;},M_{\text{mu}},M_{\text{cnv}}\right\}\in R^{m\times 706}$ to represent the multiomics data associated with 706 genes in these cell lines (see Supplementary Fig. S1). In addition, based on known IC50 values between drugs and cell lines, we construct a cell line-drug IC50 values matrix for cell lines. The matrix can be described as $M_{\text{CD}}\in R^{m\times n}$. We can get the cell line similarity matrix ${\text{SM}}_{\text{CD}}\in R^{m\times m}$ calculated by Chebyshev distance. We denote $v^{a}$ and $v^{b}$ to represent the *a*-th and *b*-th row of a matrix, respectively. The Chebyshev distance between the *a*-th and the *b*-th row is defined as follows: where $v_{c}^{a}$ and$v_{c}^{b}$ represent the *c*-th element of $v^{a}$ and $v^{b}$, respectively. By calculating the Chebyshev distance between the rows in the matrix, we can derive a similarity matrix.


(1) $$D_{\text{Chebyshev}}(a,b)=\text{max}(\left|v_{c}^{a}-v_{c}^{b}\right|),$$

### 2.3 Drug features

Let $S_{\text{drug}}=\left\{d_{1},d_{2},\ldots d_{n}\right\}$ represent the set of all *n* different drugs. To obtain a rich set of drug features, we collect SMILES sequences of drugs, drug–drug combination scores, known drug–target interactions, known drug–disease associations, known drug–microRNA associations and known drug–adverse drug reaction (ADR) associations. Based on these drug multisource data and known IC50 values between drugs and cell lines, we construct 12 matrices for drugs. Firstly, we construct six molecular fingerprint matrices based on molecular fingerprints and the dimensions of the row in these matrices are 1024, 881, 2048, 200, 2586, and 315, respectively. Then we construct six association matrices based on the associations/interactions between drugs and biological entities and the dimensions of the row in these matrices are *n*, 822, 5181, 636, 4693, and *m*, respectively. In a molecular fingerprint or association matrix, a row represents the molecular fingerprint representation of a drug or the associations/nonassociations between the drug and a class of biological entities. Assuming that similar drugs may produce similar reactions, we calculate 12 drug similarity matrices of n×n dimensions by Chebyshev distance based on six molecular fingerprint matrices and six association matrices of drugs (see Supplementary Fig. S1). In addition, to effectively integrate the similarity information from multiple biological data sources, we use an SNF algorithm to fuse 12 similarity matrices of drugs into a fusion similarity matrix of n×n dimensions. In each similarity matrix or fusion matrix, the row represents a type of similarity vector for a drug and the value of *i*-th row and *j*-th column represents the similarity between $d_{i}$ and $d_{j}$.

#### 2.3.1 Drug fingerprints

We obtain the SMILES sequence of the drugs from PubChem database (Kim *et al.* 2019). Here, we calculate six molecular fingerprints for drugs, including Extended-Connectivity FingerPrints (ECFP), PubChem Substructure FingerPrints (PSFP), Daylight FingerPrints (DFP), RDKit 2D normalized FingerPrints (RDKFP), Explainable Substructure Partition FingerPrints (ESPFP), and Extended Reduced Graph FingerPrints (ERGFP). We construct six matrices $\{M_{\text{RDKFP}},M_{\text{ESPFP}},M_{\text{ERGFP}},M_{\text{ECFP}},M_{\text{PSFP}},M_{\text{DFP}}\}\in R^{n\times n}$ for drugs to represent the above molecular fingerprints and through Chebyshev distance to calculate six similarity matrices $\{{\text{SM}}_{\text{RDKFP}},{\text{SM}}_{\text{ESPFP}},{\text{SM}}_{\text{ERGFP}},{\text{SM}}_{\text{ECFP}},{\text{SM}}_{\text{PSFP}},{\text{SM}}_{\text{DFP}}\}\in R^{n\times n}$.

#### 2.3.2 Chemical–chemical combined scores

Some literatures show that the interaction pattern between drugs is an important information for drug response prediction (Duan 2010). We collect drug–drug combined scores from STITCH database (Kuhn *et al.* 2008). Firstly, we use PubChem compound id to map the compound id in STITCH database, which provides a large number of known and predicted interactions between compounds. Then, we collect the combined scores between drugs from STITCH database and construct $M_{\text{combined}}\in R^{n\times n}$. Since the chemical–chemical combined scores in STITCH range from 1 to 1000, we divide scores by 1000 to ensure that the similarity values of drugs are between 0 and 1. Finally, we construct the matrix ${\text{SM}}_{\text{combined}}\in R^{n\times n}$ to represent the combined scores between pair-wise drugs, if the drug–drug combined scores are known. The corresponding value in the matrix ${\text{SM}}_{\text{combined}}$ is combined scores divided by 1000, otherwise it is set to 0.

#### 2.3.3 Drug–target interactions

The known drug–target interactions are obtained from DrugBank database (Wishart *et al.* 2018) and DGIdb database (Freshour *et al.* 2021). Firstly, we find that the id of drugs through PubChem database. Then, we use the PubChem id to map the drugs in DrugBank database and obtain the known interactions between drugs and targets. In addition, we download known drug–target interactions from DGIdb database and use drug name to map the drugs. After screening and integration, there are 822 targets that have interactions with the drugs of the benchmark datasets. We use the matrix $M_{\text{target}}\in R^{n\times 822}$ to represent the known interactions of drugs in the benchmark dataset and 822 targets. If the drug interacts a target, the corresponding value of the matrix $M_{\text{target}}$ is set to 1, otherwise, it is set to 0. The similarity matrix calculated by Chebyshev distance can be described as ${\text{SM}}_{\text{target}}\in R^{n\times n}$.

#### 2.3.4 Drug–disease associations

It is reported that the relationships between drugs and diseases are predictive of drug-related prediction tasks. We obtain the known drug–disease associations from CTDbase database (Davis *et al.* 2021), which provides a vast array of associations between drugs, genes, diseases, and more. We download the known drug–disease associations from CTDbase and use the names of drugs in the benchmark dataset to map the associations. After screening, there are 5181 diseases associated with the drugs in the benchmark dataset. We use the matrix $M_{\text{disease}}\in R^{n\times 5181}$ to store the known associations between drugs in the benchmark dataset and 5181 diseases. If the drug associates with a disease, the corresponding value of the matrix $M_{\text{disease}}$ is set to 1, otherwise, 0. We use the matrix ${\text{SM}}_{\text{disease}}\in R^{n\times n}$ to represent the similarity matrix calculated by Chebyshev distance.

#### 2.3.5 Drug–microRNA associations

It is documented that microRNA pharmacogenomics facilitates the understanding of different individual responses to certain drugs. We obtain the known drug–microRNA associations from ncDR database (Dai *et al.* 2017), which provides some validated and predicted drug resistance-associated microRNAs and long coding RNAs. We download the known drug–microRNA associations and use the drug name to map the drugs in ncDR database. After screening, the number of microRNA associated with the drugs in the benchmark dataset is 636. We use the matrix $M_{\text{miRNA}}\in R^{n\times 636}$ to represent the known drug–microRNA associations. If the drug is associated with a type of microRNA, the corresponding value of the matrix $M_{\text{miRNA}}$ is set to 1, otherwise, it is set to 0. The similarity matrix calculated by Chebyshev distance is described as ${\text{SM}}_{\text{miRNA}}\in R^{n\times n}$.

#### 2.3.6 Drug–ADR associations

We collect the drug–ADR associations from SIDER (Kuhn *et al.* 2010) and ADReCS (Cai *et al.* 2015) databases. SIDER contains records of marketed drugs and their adverse drug reactions. ADReCS is a comprehensive ADR ontology database, containing 1355 single active ingredient drugs and 134 022 drug–ADR pairs. We download known drug–ADR associations from these databases and use PubChem compound id to map the drugs. After screening, the number of ADR associated with drugs of the benchmark datasets is 4693. We use the matrix $M_{\text{ADR}}\in R^{n\times 4693}$ to represent the known drug-side effect associations. If the drug associates with a type of ADR, the corresponding value of the matrix is set to 1, otherwise, it is set to 0. We use the matrix ${\text{SM}}_{\text{ADR}}\in R^{n\times n}$.

#### 2.3.7 Drug interaction profiles

Similar to the cell lines, based on the transposition of the IC50 value matrix of cell line–drug pairs, we can calculate the similarity matrix ${\text{SM}}_{\text{DC}}\in R^{n\times n}$ for drugs by Chebyshev distance.

#### 2.3.8 The completion and fusion of drug similarity matrices

Since our method introduces multiple biological data sources of drugs, the record of these biological data sources is incomplete, resulting in sparse association matrices. Considering the above limitations, we use an SNF algorithm to fuse the similarity matrices calculated based on drug SMILES sequences into a fusion similarity matrix and then use it to fill the similarity matrices calculated based on the drug related biological entities. More specifically, firstly, since each SMILES sequence corresponds to a unique chemical structure, the similarity matrices calculated based on SMILES sequences are dense and complete and can accurately represent the feature of drug structures. SNF algorithm can exploit the complementarity of data to compute and fuse similarity networks obtained from each type of data separately (Wang *et al.* 2014). Here we use the SNF algorithm to fuse six similarity matrices calculated based on drug SMILES sequences (${\text{SM}}_{\text{ECFP}}$, ${\text{SM}}_{\text{PSFP}}$, ${\text{SM}}_{\text{DFP}}$, ${\text{SM}}_{\text{RDKFP}}$, ${\text{SM}}_{\text{ESPFP}}$ and ${\text{SM}}_{\text{ERGFP}}$) into a fused matrix ${\text{DM}}_{\text{SMILES}}^{\prime }\in R^{n\times n}$. Secondly, since the associations/interactions between biological entities and drugs are noisy, the similarity matrices calculated based on these matrices are incomplete. Therefore, we use ${\text{SM}}_{\text{SMILES}}^{\prime }$ to fill the five similarity matrices calculated based on the drug-related biological entities. We define the set $S_{D}=\left\{M_{\text{combined}},M_{\text{target}},M_{\text{disease}},M_{\text{miRNA}},M_{\text{ADR}}\right\}$, the set $S_{\text{DM}}=\left\{{\text{SM}}_{\text{combined}},{\text{SM}}_{\text{target}},{\text{SM}}_{\text{disease}},{\text{SM}}_{\text{miRNA}},{\text{SM}}_{\text{ADR}}\right\}$ and the set $S_{\text{DM}}^{\prime }=\left\{{\text{SM}}_{\text{combined}}^{\prime },{\text{SM}}_{\text{target}}^{\prime },{\text{SM}}_{\text{disease}}^{\prime },{\text{SM}}_{\text{miRNA}}^{\prime },{\text{SM}}_{\text{ADR}}^{\prime }\right\}$. The filling process can be described as the following: where $S_{\text{DM}}[d](r)$ and $S_{\text{DM}}^{\prime }[d](r)$ represent the *r*-th row and *r*-th column of the *d*-th element in $S_{\text{DM}}$ and $S_{\text{DM}}^{\prime }$, respectively. ${\text{SM}}_{\text{SMILES}}(r)$ represents the *r*-th row and *r*-th column in ${\text{SM}}_{\text{SMILES}}$.


(2) $$S_{\text{DM}}^{\prime }[d](r)=\{\begin{array}{cc} {\text{SM}}_{\text{SMILES}}(r) & \text{if}\;\text{the}\;r-\text{th}\;\text{row}\;\text{in}\;S_{D}[d]\;\text{is}\\ \text{the}\;\text{zero}\;\text{vector} & \\ S_{\text{DM}}[d](r) & \;\text{otherwise}\; \end{array},$$

### 2.4 Method

**Figure 1.**
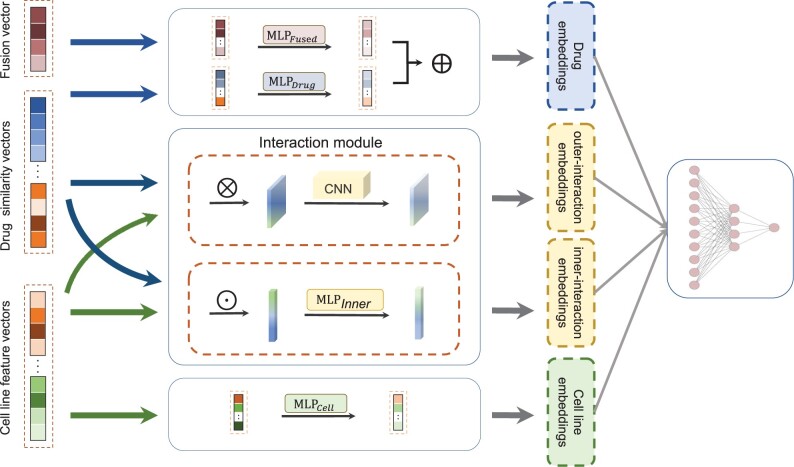
The architecture of MSDRP

After data preprocessing, we can obtain 12 similarity matrices (${\text{SM}}_{\text{combined}}^{\prime }$, ${\text{SM}}_{\text{target}}^{\prime }$, ${\text{SM}}_{\text{disease}}^{\prime }$, ${\text{SM}}_{\text{miRNA}}^{\prime }$, ${\text{SM}}_{\text{ADR}}^{\prime }$, ${\text{SM}}_{\text{ECFP}}$, ${\text{SM}}_{\text{PSFP}}$, ${\text{SM}}_{\text{DFP}}$, ${\text{SM}}_{\text{RDKFP}}$, ${\text{SM}}_{\text{ESPFP}}$, ${\text{SM}}_{\text{ERGFP}}$ and ${\text{SM}}_{\text{DC}}$) for drugs and four matrices ($M_{\;\text{exp}\;}$,$M_{\text{mu}}$,$M_{\text{cnv}}$ and ${\text{SM}}_{\text{CD}}$) for cell lines. To effectively integrate the similarity information from multiple biological data sources, we also use an SNF algorithm to fuse 12 similarity matrices of drugs into a fusion similarity matrix ${\text{SM}}_{\text{fusion}}\in R^{n\times n}$. Motivated by effectively joint effect of drug molecular structures and the associations/interactions between drugs and biological entities in drug-related prediction tasks (Zhao *et al.* 2022), we propose a novel deep learning model, called MSDRP, for drug response prediction based on multisource data of drugs and cell lines, respectively. We take drug–cell line pair $d_{i}$-$c_{j}$ as an example. For $d_{i}$, we extract the *i*-th row of each similarity matrix and the fusion matrix to generate 12 similarity vectors and a fusion vector, respectively. Similarly, for $c_{j}$, we extract the *j*-th row of each feature matrix to generate four cell line feature vectors. The model can be described as four steps (see Fig. 1): (i) projecting the 12 similarity vectors of the $d_{i}$ and four feature vectors of $c_{j}$ into the vector space of the same dimension; (ii) capturing $d_{i}$ and $c_{j}$ interaction embeddings through the interaction module; (iii) extracting embeddings of $d_{i}$ and embeddings of $c_{j}$ through the ${\text{MLP}}_{\text{Fused}}$ module, ${\text{MLP}}_{\text{Drug}}$ module, and ${\text{MLP}}_{\text{Cell}}$ module; and (iv) integrating higher-order correlation embeddings of $d_{i}$-$c_{j}$, embeddings of $d_{i}$ and embeddings of $c_{j}$, and then feed into a prediction module to predict the IC50 values. Next, we discuss the implementation details of each step.

In step 1, for similarity vectors of $d_{i}$, we design 12 transformation matrices, i.e.$\left\{G_{1},G_{2},\ldots ,G_{12}\right\}\in R^{n\times s}$ where *s* is set to 128, representing the dimension of each vector transformed into a specific vector space. The transformed vector $g_{k}^{\prime }$ of *k*-th similarity vector of $d_{i}$ can be calculated as follows: where $g_{k}$ is the *k*-th similarity vector of $d_{i}$. Similarly, for feature vectors of $c_{j}$, we design four transformation matrices $\left\{H_{1},H_{2},H_{3},H_{4}\right\}$. The transformed vector $h_{l}^{\prime }$ of the *l*-th feature vector of $c_{j}$ can be calculated as follows: where $h_{l}$ is the *l*-th feature vector of $c_{j}$.


(3) $$g_{k}^{\prime }=g_{k}G_{k},$$

(4) $$h_{l}^{\prime }=h_{l}H_{l},$$

In step 2, we design an interaction module, including both outer product and inner product units, to capture fine-grained and coarse-grained interactions between $d_{i}$ and $c_{j}$. In outer product unit, we first perform the outer product operation between the transformed vectors of $d_{i}$ and the transformed vectors of $c_{j}$. For the *k*-th transformed vector $g_{k}^{\prime }$ of $d_{i}$ and the *l*-th transformed vector $h_{l}^{\prime }$ of $c_{j}$, the outer-interaction map ${\text{Intermap}}_{k,l}^{\text{outer}}$ can be calculated as follows: where ⊗ represents the outer product operation and ${\text{Intermap}}_{k,l}^{\text{outer}}$ is an s×s matrix. Here, we can get 48 (12×4) different outer-interaction maps to represent $d_{i}$-$c_{j}$. Then we use the CNN network to learn the outer-interaction embeddings from the multiple outer-interaction maps. The CNN network consists of two residual blocks and a CNN layer. The residual block can be described as follows:


(5) $${\text{Intermap}}_{k,l}^{\text{outer}}=g_{k}^{\prime }⊗h_{l}^{\prime },$$

(6) $$x_{e+1}=h(x_{e})+\tau (x_{e},W_{e}).$$

The residual block is divided into two parts direct mapping part $h(x_{e})$ and residual part $\tau (x_{e},W_{e})$. The $x_{e+1}$ is the *e*-th layer output. The CNN network can effectively utilize the local features of $d_{i}$ and $c_{j}$ interactions. Finally, we use a max-pooling layer to capture the global information from the extracted interaction embeddings. In inner product unit, we first perform the inner product operation and calculate the inner-interaction vectors as follows: where ⊙ represents the inner product operation and ${\text{Intermap}}_{k,l}^{\text{inner}}$ is a vector of dimension *s*. Here, we can get 48 (12×4) different inner-interaction vectors to represent $d_{i}$-$c_{j}$. Then we use an ${\text{MLP}}_{\text{Inner}}$ module to obtain the inner-interaction embeddings of $d_{i}$-$c_{j}$ from the multiple inner-interaction vectors. The ${\text{MLP}}_{\text{Inner}}$ module consists of four fully connected layers, in which the numbers of neurons are 1024, 1024, 512, and 128, respectively.


(7) $${\text{Intermap}}_{k,l}^{\text{inner}}=g_{k}^{\prime }⊙h_{l}^{\prime },$$

In step 3, here we use three similar MLP modules to capture the embeddings of $d_{i}$ and $c_{j}$. We firstly use the ${\text{MLP}}_{\text{Fused}}$ module and the ${\text{MLP}}_{\text{Drug}}$ module to jointly learn embeddings of $d_{i}$ from the fusion vector and the transformed vectors, respectively. Then, we use the ${\text{MLP}}_{\text{Cell}}$ module to learn embeddings of $c_{j}$ from the transformed vectors. Each MLP has two fully connected hidden layers, and the number of neurons in hidden layer is *s*. The activation function in each layer in MLP modules is the Rectified Linear Unit (ReLU) function.

In step 4, we concatenate higher-order correlation embeddings of $d_{i}$-$c_{j}$, embeddings of $d_{i}$ and embeddings of $c_{j}$, and then feed them into a prediction module consisting of four fully connected layers to produce the final predicted IC50 values between $d_{i}$ and $c_{j}$.

To train our model, we use the PyTorch (Paszke *et al.* 2019) framework to implement the model code. The model is trained end-to-end using the mean square error as the loss function. We use the Adam (Jais *et al.* 2019) as the optimizer with the default learning rate of 1e-4 and the weight decay coefficient of 3e-4. We perform early stopping to avoid overfitting. If the loss of models on the validation set does not decrease within 10 epochs, the training will stop.

## 3 Results

### 3.1 Performance evaluation metrics and framework

We first compare our model with some state-of-the-art methods based on the benchmark dataset. We split the benchmark dataset into nonoverlapping training, validation, and testing sets in a ratio of 8:1:1. For comparison fairness, the hyperparameters in other methods are set according to the optimal value as suggested by the authors. To further demonstrate the effectiveness of integrating multisource features, we also add our five drug-related biological entities associations/interactions to TGSA (TGSA+). We add a fully connected layer to extract features from these associations/interactions and then concatenate it with the latent representations of drugs output by the GNN module in TGSA to construct new representations of drugs. We use three indicators widely used in regression tasks to measure performance: root mean square error (RMSE), mean absolute error (MAE), and Pearson correlation coefficient (*r*). Table 1 shows the comparison results between MSDRP and other methods. Our method gets 5.02%, 3.71%, and 0.3% improvements in RMSE, MAE, and *r* over the second-best method TGSA, respectively. The comparison result indicates that the introduction of known associations/interactions between drugs and biological entities as the drug features and the capture of interactions between drugs and cell lines through the interaction module can effectively improve the performance of the models in drug reaction prediction. Moreover, we compare the prediction performance of different distance calculation methods (see Supplementary Table S1) and select Chebyshev distance as the better similarity measure to calculate the similarity matrices. In addition, we conduct the ablation experiment to observe whether each module is beneficial to MSDRP, and the results show that the current model architecture and feature selection scheme are optimal for our prediction tasks (see Supplementary Table S2).

**Table 1.** Comparison between our model and some state-of-the-art models.

Methods	RMSE	MAE	*r*
tCNNs	0.951±0.009	0.700±0.008	0.942±0.001
DeepCDR	0.914±0.018	0.674±0.014	0.946±0.001
GraphDRP	0.953±0.019	0.702±0.017	0.942±0.002
BiGPicture	1.248±0.008	0.997±0.006	0.742±0.001
Precily	1.353±0.009	1.000±0.009	0.879±0.003
TGSA	0.877±0.008	0.646±0.006	0.951±0.001
TGSA+	0.868±0.012	0.639±0.010	0.952±0.002
GADRP	0.962±0.010	0.719±0.009	0.916±0.001
MSDRP	**0.833** ± **0.005**	**0.622** ± **0.007**	**0.954** ± **0.001**

The optimal value in each column has been emphasized in bold.

### 3.2 *De novo* test and independent test

To evaluate the performance of our model for new drug response prediction, we conduct *de novo* test with two experiment settings for a comprehensive comparison. Assuming that $D_{\text{train}}$ and $C_{\text{train}}$ are the sets of drugs and cell lines in the training set, respectively. When predicting the drug response between drug $d_{i}$ and cell line $c_{i}$ in the testing set, there are two different experimental settings:

1.
${\text{ES}}_{1}$ : There are no drug $d_{i}$ and known IC50 values of drug $d_{i}$ (all related-cell lines are unknown) in the training set: $d_{i}\notin D_{\text{train}}$.
2.
${\text{ES}}_{2}$ : There are no cell $c_{i}$ and known IC50 values of cell $c_{i}$ (all related-drugs are unknown) in the training set: $c_{i}\notin C_{\text{train}}$ .

In *de novo* test, we evaluate tCNNS, DeepCDR, MSDRP, BiGPicture, Precily, and TGSA under ${\text{ES}}_{1}$ and ${\text{ES}}_{2}$ settings on the benchmark dataset, respectively. We randomly select 20% drugs/cell lines in the benchmark dataset and the ratio of drugs and cell lines under ${\text{ES}}_{1}$ and ${\text{ES}}_{2}$ settings in the test set, validation set, and training set is 4:1:1, respectively. Tables 2 and 3 show the results under ${\text{ES}}_{1}$ and ${\text{ES}}_{2}$ experimental settings, respectively.

**Table 2.** Comparison results of the proposed model and other methods on the benchmark dataset under the setting ${\text{ES}}_{1}$.

Methods	RMSE	MAE	*r*
tCNNs	1.829±0.012	1.534±0.009	0.654±0.002
DeepCDR	1.831±0.023	1.663±0.016	0.633±0.003
BiGPicture	1.741±0.016	1.254±0.009	0.852±0.001
Precily	1.527±0.007	1.133±0.007	0.860±0.002
TGSA	1.794±0.009	1.301±0.008	0.839±0.002
GADRP	1.480±0.011	1.369±0.010	0.833±0.001
MSDRP	**1.285** ± **0.009**	**0.901** ± **0.008**	**0.915** ± **0.001**

The optimal value in each column has been emphasized in bold.

**Table 3.** Comparison results of the proposed model and other methods on the benchmark dataset under the setting ${\text{ES}}_{2}$.

Methods	RMSE	MAE	*r*
tCNNs	1.429±0.008	1.006±0.007	0.822±0.002
DeepCDR	1.575±0.013	1.194±0.010	0.688±0.001
BiGPicture	1.276±0.010	1.010±0.005	0.892±0.003
Precily	1.405±0.008	1.049±0.009	0.877±0.002
TGSA	1.344±0.009	1.039±0.012	0.875±0.001
GADRP	1.255±0.011	1.069±0.009	0.717±0.002
MSDRP	**1.024** ± **0.009**	**0.772** ± **0.006**	**0.920** ± **0.001**

The optimal value in each column has been emphasized in bold.

In addition, we use the independent test to show the predictive performance of our method. Our independent dataset contains 763 IC50 values including 12 drugs and 138 cell lines. Here, we compare MSDRP with BiGPicture, Precily, and TGSA on independent dataset and Table 4 shows the results.

**Table 4.** Comparison results of our model and other methods on the independent dataset.

Methods	RMSE	MAE	*r*
tCNNs	1.942	1.461	0.606
DeepCDR	1.892	1.358	0.674
BiGPicture	1.805	1.279	0.732
Precily	1.932	1.479	0.595
TGSA	1.847	1.321	0.656
GADRP	1.880	1.569	0.533
MSDRP	**1.644**	**1.067**	**0.847**

The optimal value in each column has been emphasized in bold.

These results show that our model is competitive and has better performance than three state-of-the-art deep learning models.

### 3.3 Analysis of the contribution of each feature of drug and cell line

One of the main advantages of our framework is the use of data from multiple data sources. To build MSDRP, we construct 12 feature matrices and four feature matrices for drugs and cell lines, respectively. Next, we investigate the matrices that produce the most contribution to MSDRP and the consistency and complementarity of these different matrices. To answer the first questions, we delete a matrix of drug or cell line in turn and use the remaining 11 matrices of drugs or three matrices of cell lines to represent the features of drugs or cell lines, and then reconstruct an MSDRP. As a result, 16 MSDRP models based on different combinations of data sources are obtained by using the same hyperparameters mentioned in Section 2. Table 5 shows the performance when one drug feature matrix is removed. For drugs, MSDRP without ${\text{SM}}_{\text{ESPFP}}$ produces the highest RMSE and MAE. Table 6 shows the performance of our method when one cell line feature matrix is removed. For cell lines, without $M_{\;\text{exp}\;}$ produce the highest RMSE and MAE.

**Table 5.** Performance of our method when one drug feature matrix is removed.

Excluded matrix	RMSE	MAE	*r*
${\text{SM}}_{\text{DFP}}$	0.850	0.629	0.953
${\text{SM}}_{\text{ERGFP}}$	0.855	0.636	0.952
${\text{SM}}_{\text{ESPFP}}$	0.868	0.642	0.951
${\text{SM}}_{\text{ECFP}}$	0.848	0.630	0.953
${\text{SM}}_{\text{PSFP}}$	0.858	0.636	0.952
${\text{SM}}_{\text{RDKFP}}$	0.847	0.629	0.953
${\text{SM}}_{\text{target}}^{\prime }$	0.853	0.635	0.952
${\text{SM}}_{\text{ADR}}^{\prime }$	0.855	0.633	0.952
${\text{SM}}_{\text{disease}}^{\prime }$	0.857	0.642	0.952
${\text{SM}}_{\text{miRNA}}^{\prime }$	0.861	0.642	0.951
${\text{SM}}_{\text{combined}}^{\prime }$	0.854	0.631	0.952
${\text{SM}}_{\text{DC}}$	0.859	0.637	0.951

**Table 6.** Performance of our method when one cell line feature matrix is removed.

Excluded matrix	RMSE	MAE	*r*
$M_{\;\text{exp}\;}$	0.863	0.641	0.951
$M_{\text{mu}}$	0.858	0.646	0.953
$M_{\text{cnv}}$	0.851	0.634	0.953
$M_{\text{CD}}$	0.853	0.638	0.951

Therefore, these contribute the most to the model. To answer the second questions, we plot the correlation heatmaps for the feature matrices of drugs and cell lines, respectively. Specifically, we calculate the Spearman correlation coefficients between the 12 feature matrices of the drug and the four feature matrices of the cell line, respectively. Then we plot two heatmaps based on the calculated correlation coefficient matrices to represent the correlation between multiple features of the drug and cell line, respectively. In addition, to explore the consistency and complementarity of these multisource data, we compute the Pearson correlation coefficients for all the feature pairs and plot two heatmaps. As shown in Supplementary Figs S2 and S3, there is complementary information in different data, and the combination of these different data is beneficial in enhancing the predictive performance of our model.

### 3.4 Analysis of the association between drugs and pathways

**Figure 2.**
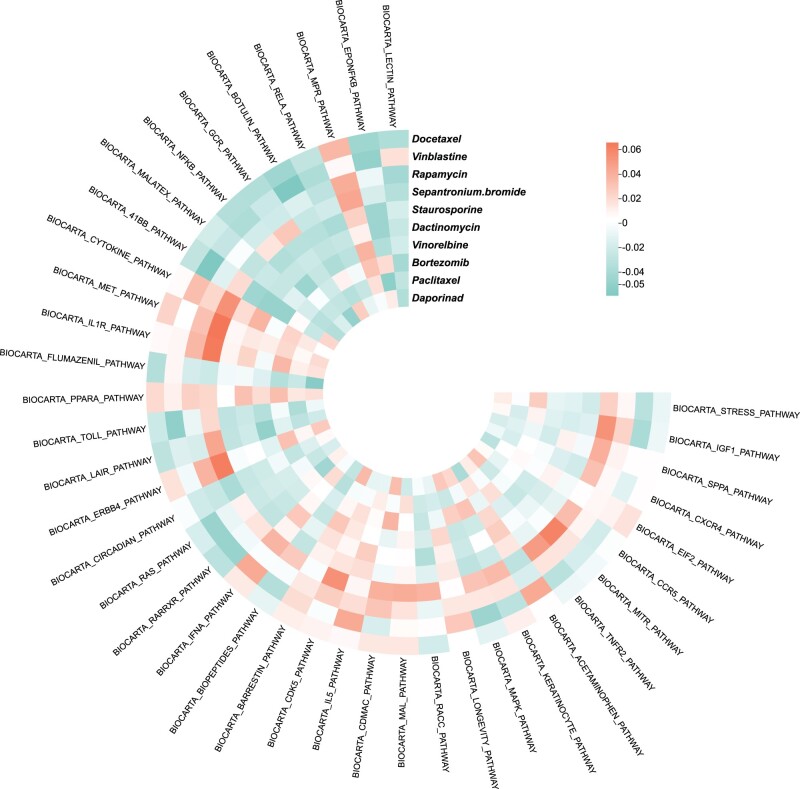
Association of the 10 drugs with the pathways. For visualization, the top 40 pathways with the highest cross-drug correlations are selected. Negative and positive correlations between pathway activity and drug sensitivity scores are denoted as “sensitive” and “resistant” associations, respectively

Some literature shows that drugs exert the effects by affecting related biological pathways rather than targeting a single protein (Wang *et al.* 2021). To analyze associations between drugs and signaling pathways, we obtain 292 Biocarta pathway gene sets from MSigDB (Liberzon *et al.* 2011). The MSigDB database provides many gene sets. A gene set is called a pathway which is a collection of genes with similar positions or functions. We obtain gene expression data of 580 cell lines in our benchmark dataset from CCLE database. Based on gene expression data and gene sets, we calculate pathway activity scores for each cell line, following the method of Suphavilai *et al.* (2018). We predict IC50 values between 170 drugs and 580 cell lines, and then select the top 10 drugs with the lowest average of predicted IC50 values. We then calculate the Pearson correlation between the pathway activity scores and the predicted drug responses (see Fig. 2). We observe that the drugs with the lowest average of predicted IC50 values are sensitive to most of the key pathways, which indicate that our model can accurately predict drug responses. For example, most pathways are sensitive to Paclitaxel, which is consistent with the existing literature (Singla *et al.* 2002). Some studies show that inhibition of JNK (one of the main four groups of the MAPK pathway) or the absence of JNK prevents vinblastine-induced cell death (Kolomeichuk *et al.* 2008),which is consistent with our prediction that the MAPK pathway is sensitive to vinblastine. These results highlight the capability of MSDRP for discovering drug sensitivity and its interpretability. In addition, we conduct the case analysis on the top three drugs with the lowest average of predicted IC50 values. For each drug, MSDRP estimates the predicted IC50 values for all cell lines. We rank the predicted IC50 values and select the top 10 cell lines for drugs. We find that many newly predicted drug responses are supported by DrugBank database and recently published experimental studies (see Supplementary Table S3).

### 3.5 Analysis of drug response in AML cell lines

**Figure 3.**
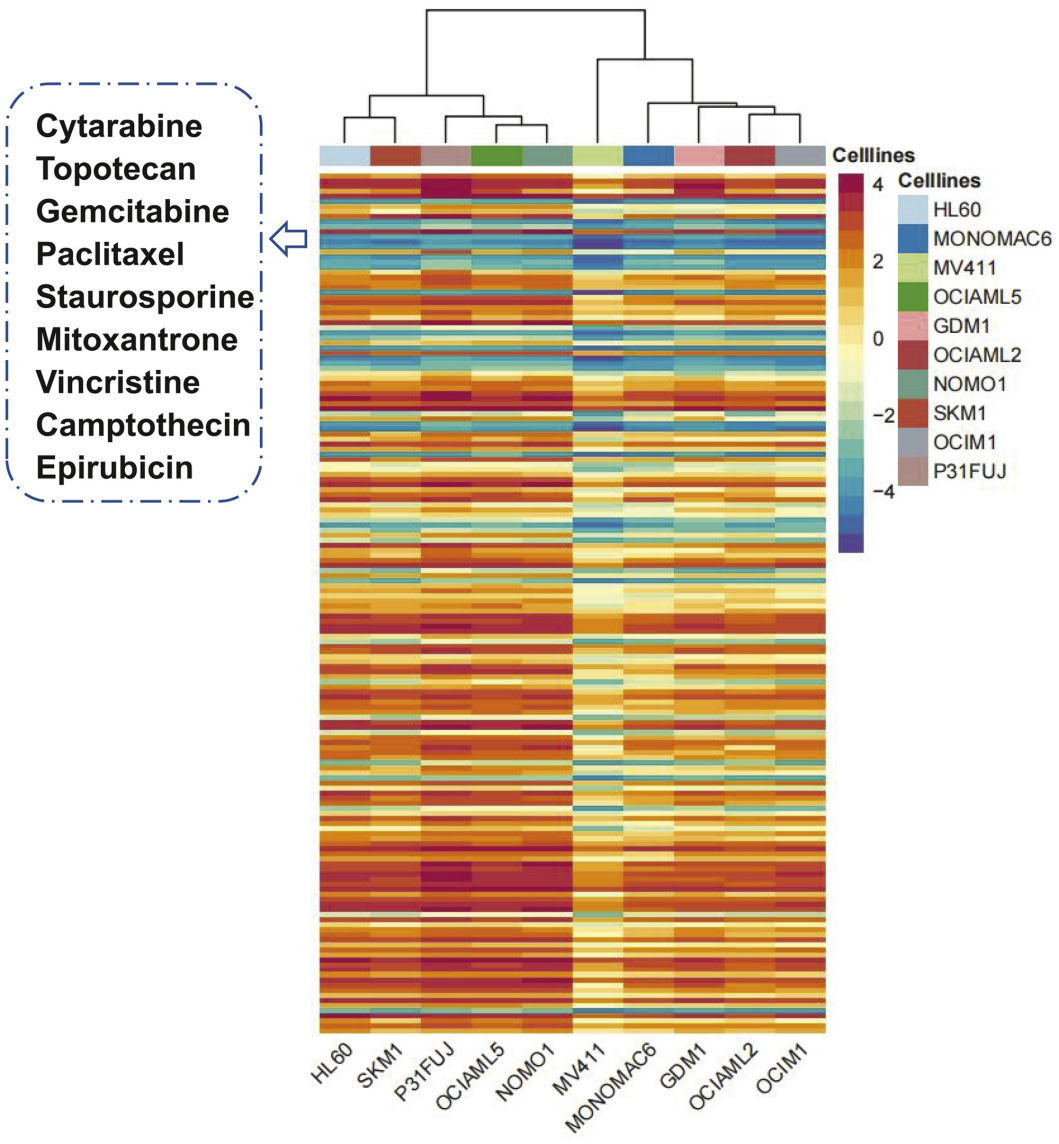
Heatmap of drug response of 170 drugs on 10 samples

Acute myeloid leukemia (AML) is a cancer caused by the excessive proliferation of blood cells in the bone marrow, which is characterized by drug resistance, relapse, and refractory, etc. (Khwaja *et al.* 2016). Although some useful drugs are developed to the treatment of AML, new therapy options are urgently needed to further improve the survival rate of patients. We select AML cell lines from our dataset and perform *de novo* test for these cell lines as one of the case studies. More specifically, we first select 10 AML cell lines in the benchmark dataset and then remove the drug responses associated with these cell lines from the benchmark dataset and use the remaining drug responses as the training set. Finally, we predict IC50 values between 170 drugs and these cell lines and plot a heat map. Through the analysis of the prediction results shown in Fig. 3, we find that nine drugs are sensitive to these AML cell lines and some biological experiments show that the above nine drugs can play a therapeutic role in AML (Beguin *et al.* 1997, Cortes *et al.* 2002, Kindler *et al.* 2005, Yu *et al.* 2007, Advani *et al.* 2010), which demonstrates that MSDRP can be used as a powerful tool for predicting drug response.

To justify the reliability of the drug representations based on similarity calculation, we focus on analyzing the correlation of the features between these drugs. We first screen 12 types of features of these nine drugs from feature matrices. Then we calculate the correlations among multiple features of these drugs separately, and plot 12 correlations heatmaps of features (Supplementary Fig. S4). From the results, we can find that there is at least one correlation evidence between these drugs. Among them, eight of the 12 features between Cytarabine and Gemcitabine are highly correlated (as shown in Table 7). These results justify the use of similarity to represent drugs and the significantly positive effect of these similarities on the model correctly predicting IC50 values between drugs and cell lines.

**Table 7.** Numbers of highly correlation evidences among nine drugs.

Drug name	Drug name	The number of correlations
Cytarabine	Gemcitabine	8
Camptothecin	Topotecan	8
Mitoxantrone	Epirubicin	8
Mitoxantrone	Camptothecin	5
Mitoxantrone	Paclitaxel	5
Mitoxantrone	Epirubicin	5
Paclitaxel	Epirubicin	5
Mitoxantrone	Staurosporine	4
Mitoxantrone	Topotecan	4
Camptothecin	Epirubicin	4
Camptothecin	Staurosporine	4
Paclitaxel	Staurosporine	4
Paclitaxel	Gemcitabine	4
Epirubicin	Staurosporine	4
Epirubicin	Topotecan	4

## 4 Discussion and conclusion

In this article, we develop a new learning method to integrate multisource data of drugs and cell lines for predicting drug response. MSDRP introduces an interaction module and an SNF algorithm to integrate multisource heterogeneous data of drugs and cell lines. To verify the effectiveness of our model, we compare MSDRP with the existing state-of-the-art models. Our results show that MSDRP is superior to competing methods. Furthermore, we evaluate the performance of our model in the response prediction of new drugs through *de novo* test and independent test. Moreover, we perform case studies to illustrate the interpretability of our model and the plausibility of representing drugs using feature vectors derived from similarity calculated based on multisource data. All experimental results show that our model performs better on drug response prediction tasks compared to the existing methods.

Although MSDRP has shown effective performance in predicting drug response, it is important to be aware of several limitations. Firstly, the known associations/interactions between drugs and biological entities are incomplete, resulting in sparse association matrices. Secondly, the number of samples is critical for model training, but gathering a large number of known IC50 values between drugs and cell lines is difficult. Furthermore, since drugs are composed of molecules, it is our ideal situation to be able to represent drugs through graphs. In the future, we will further collect drug-related data and consider using GNN to capture graph-level representations of drugs.

## Supplementary Material

btad514_Supplementary_Data
