# Supplementary material for: MSDRP: a deep learning model based on multisource data for predicting drug response
Source: Bioinformatics. 2023 Aug 22;39(9):btad514. doi: 10.1093/bioinformatics/btad514 (PMC10474952; doi:10.1093/bioinformatics/btad514)
Supplement: btad514_Supplementary_Data [file btad514_supplementary_data.pdf]

Supplementary Information of "MSDRP: a deep learning model based on multi-source data for predicting drug response"

1 Comparison between different distance metrics

Supplementary Table S1. Comparison between different distance metrics.

Distance	RMSE	MAE	r
cosine similarity	0.889	0.646	0.938
euclidean distance	0.884	0.643	0.933
correlation coefficient	0.873	0.652	0.949
jaccard similarity	0.866	0.642	0.940
chebyshev distance	**0.833**	**0.622**	**0.954**

2 Ablation experiment

To illustrate the effectiveness of each component in our model, we compare our model with MSDRP removing inner product operation ($MSDRP_{no\_inner}$), MSDRP by removing outer product operation ($MSDRP_{no\_outer}$), MSDRP using an MLP module to extract the outer-interaction embeddings ($MSDRP_{no\_CNN}$) and MSDRP removing the operation of fusing drug features through the SNF algorithm ($MSDRP_{no\_SNF}$). Supplementary Table S2 shows the ablation experimental results and these results indicate the current model architecture and feature selection scheme are appropriate for our prediction task.

Supplementary Table S2. The results of ablation experiment.

	RMSE	MAE	r
$MSDRP_{no\_CNN}$	0.868	0.646	0.951
$MSDRP_{no\_outer}$	0.858	0.639	0.952
$MSDRP_{no\_inner}$	0.931	0.675	0.946
$MSDRP_{no\_SNF}$	0.847	0.631	0.953

3 Data collection and processing.

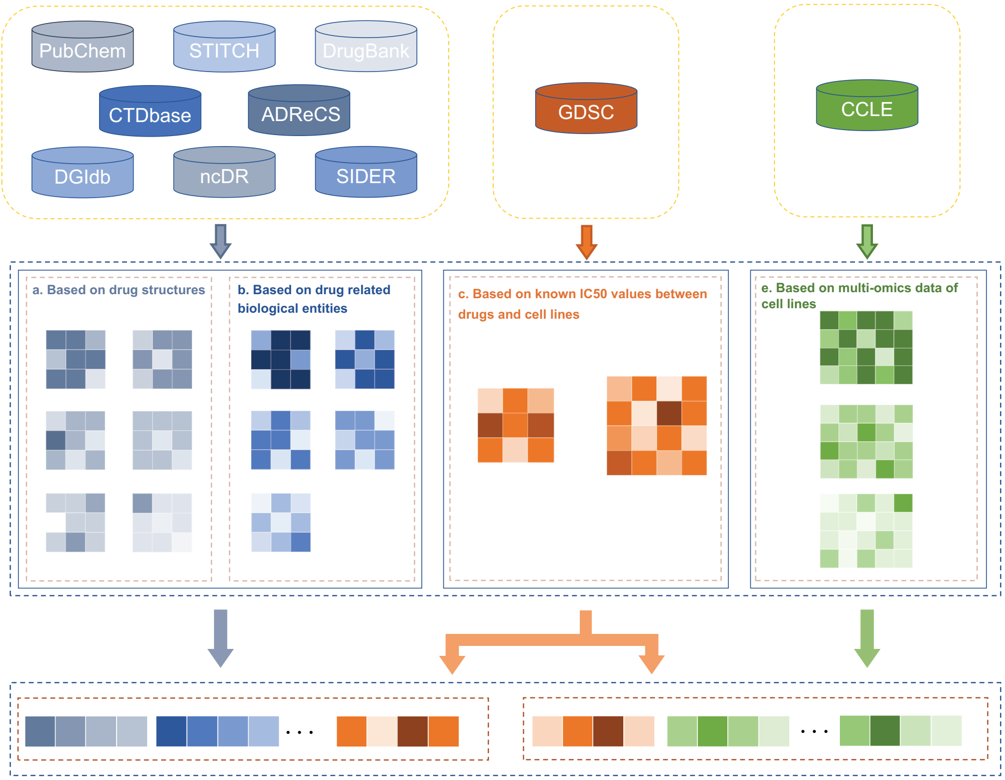

Supplementary Fig S1. Data collection and processing. Conducting 12 similarity matrices of drugs and 4 feature matrices of cell lines from 10 databases and projecting them into the vector space of the same dimension.

4 The correlation for the features of drugs and cell lines

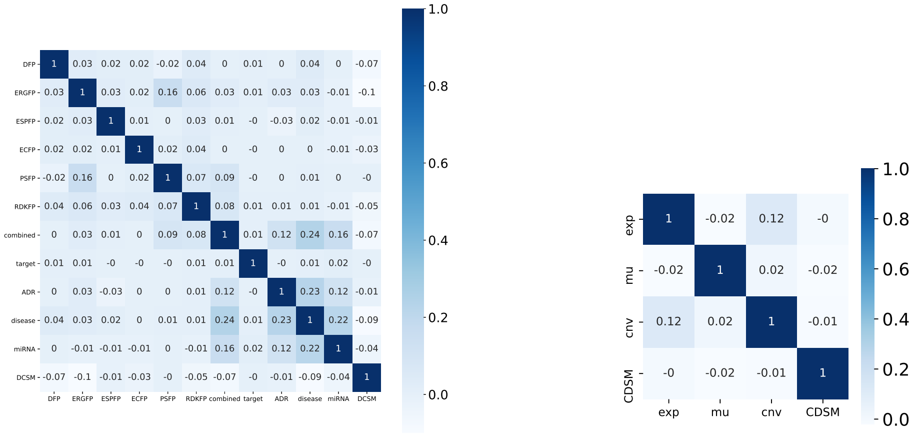

Supplementary Fig S2. The correlation heatmaps for the features of drugs and cell lines (a) the heatmaps for 12 features of drugs. (b) the heatmaps for 4 features of cell lines.

5 The correlations heatmaps of 12 features between 9 drugs

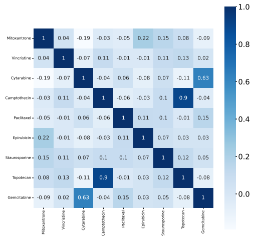

(a) DFP

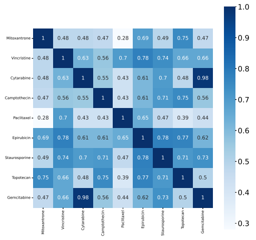

(b) ERGFP

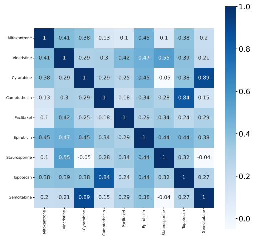

(c) ESPFP

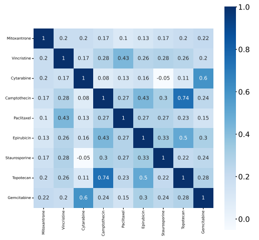

(d) ECFP

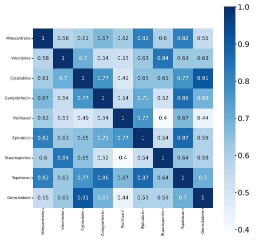

(e) PSFP

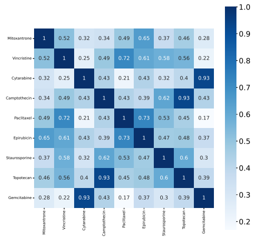

(f) RDKFP

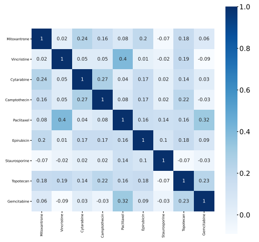

(g) known drug-target interactions

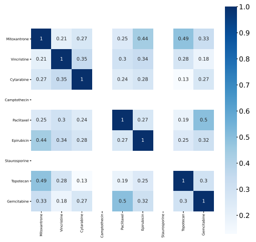

(h) known drug-ADR associations

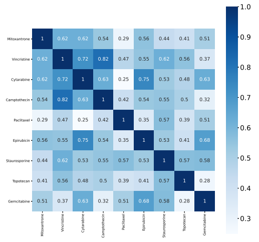

(i) known drug-disease associations

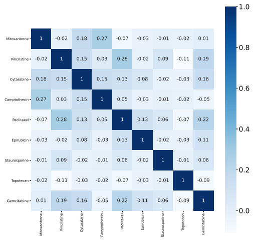

(j) known drug-microRNA associations

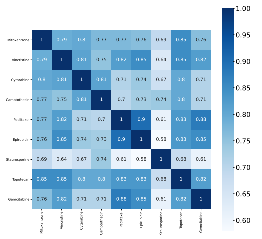

(k) drug-drug combined score

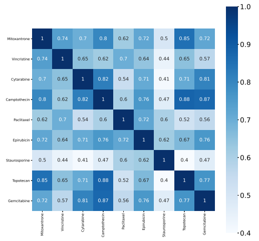

(l) drug interaction profiles

Supplementary Fig S3. the correlations heatmaps of 12 features between 9 drugs.

6 Predicting unknown drug responses

The total number of IC50 values in our benchmark dataset is 82,833, which comprises 170 drugs and 580 cell lines, with 15,767 unknown drug responses.We select the top 3 drugs with the lowest mean of predicted IC50 values, explore the top 10 cell lines which are predicted to be the most sensitive to the 3 drugs, and look for relevant evidence.

The Cancer Epigenetics Society provides several cancer epigenetics databases, including Cancer, Epigenetics, Epigenetic drugs, Cancer therapy and Cancer Epigenetics, effectively and swiftly impacts research and healthcare. medchemexpress.com is the official website of MCE (MedChemExpress), which is dedicated to providing customers with all kinds of bioactive small molecules, including inhibitors, antagonists, agonists and modulators, and classifies these into Apoptosis, cancer signaling pathways such as PI3K, etc.

Supplementary Table S3. Predicted top 10 most sensitive cell lines for top 3 drugs.

	Rank	cell line name	Description	Rank	cell line name	Description
Bortezomib	1	JURLMK1	Drugbank	6	LP1	Reference[3]
	2	SKM1	Drugbank	7	KASUMI1	Reference[4]
	3	DOHH2	Drugbank	8	KYSE510	Drugbank
	4	FARAGE	Reference[1]	9	GMS10	Drugbank
	5	OCIAML5	Reference[2]	10	HT115	Reference[5]
Daporinad	1	KE37	Drugbank	6	KOPN8	Drugbank
	2	P12ICHIKAWA	Cancer Epigenetics Society	7	SNU16	medchemexpress.com
	3	MONOMAC6	Reference[6]	8	BV173	Reference[6]
	4	ALLSIL	Drugbank	9	KCL22	Reference[6]
	5	OCIM1	Reference[7]	10	NOMO1	Reference[12]
BI-2536	1	NALM6	Reference[15]	6	NAMALWA	Reference[9]
	2	JVM3	Reference[8]	7	KOPN8	Reference[10]
	3	MOLM13	Reference[9]	8	HS578T	Reference[14]
	4	WSUNHL	Drugbank	9	BV173	selleck.cn
	5	CAL51	Reference[13]	10	RPMI-8402	Reference[11]

7 Analysis of feature correlations between drugs and cell lines

To explore the consistency and complementarity of these multi-source data, we compute the Pearson correlation coefficients for all the feature pairs and plot two heatmaps. As shown in Supplementary

Fig S4, we can ascertain that there exists complementary information within the multi-source data of drugs and cell lines. This complementarity is beneficial in enhancing the predictive performance of our model.

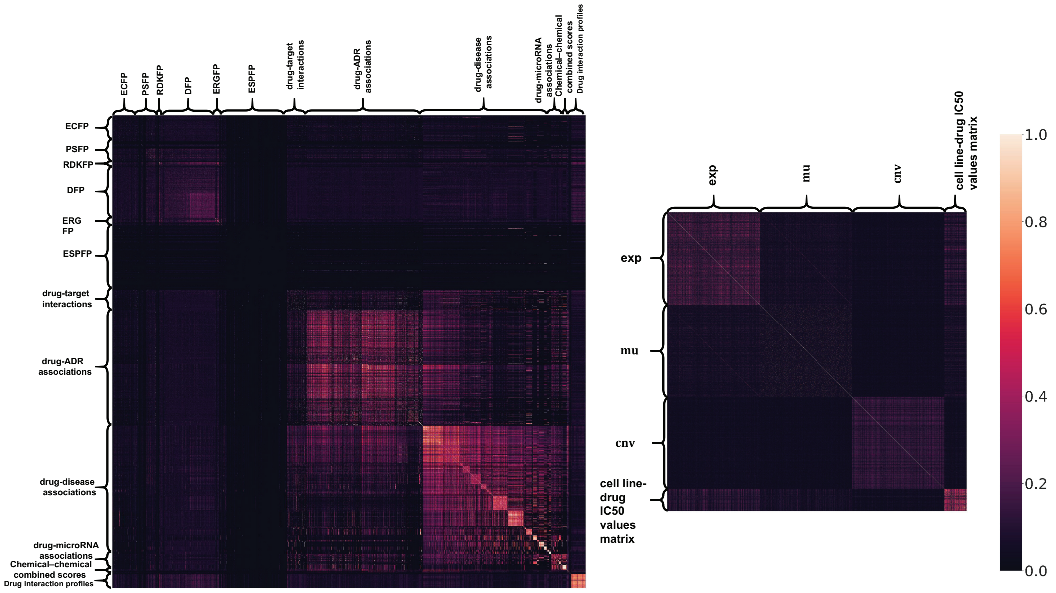

Supplementary Fig S4. The analysis of feature correlations between drugs and cell lines, respectively.

8 Analysis of the contribution of each feature of drug and cell line

This experiment involves selectively removing specific attribute matrices of drugs or feature matrices of cell lines from the model's input data. By deleting 11 attribute matrices of drugs or 3 feature matrices of cell lines one at a time, we create modified versions of MSDRP that utilize only one remaining attribute matrix of drugs or one feature matrix of cell lines to represent the corresponding features. Based on the findings presented in Supplementary Tables S4 and S5 , we can conclude that the current feature selection scheme employed in MSDRP is optimal for our prediction task. The experimental results clearly demonstrate the significance of the chosen attribute and feature matrices, thereby providing further validation of the effectiveness and stability of our model.

Supplementary Table S4. Performance of our method when 11 drug attribute matrices are removed.

Model	RMSE	MAE	r
$MSDRP\_DFP$	0.923	0.691	0.943
$MSDRP\_ERGFP$	0.883	0.663	0.948
$MSDRP\_ESPFP$	0.863	0.648	0.951
$MSDRP\_ECFP$	0.898	0.681	0.946
$MSDRP\_PSFP$	0.869	0.646	0.951
$MSDRP\_RDKFP$	0.867	0.654	0.951
$MSDRP\_target$	0.924	0.692	0.943
$MSDRP\_ADR$	0.930	0.696	0.942
$MSDRP\_disease$	0.907	0.682	0.943
$MSDRP\_miRNA$	0.883	0.652	0.947
$MSDRP\_combined$	0.912	0.674	0.942
$MSDRP\_DC$	0.916	0.687	0.941
$MSDRP$	**0.833**	**0.622**	**0.954**

Supplementary Table S5. Performance of our method when 3 cell line feature matrices are removed.

Model	RMSE	MAE	r
$MSDRP\_exp$	0.927	0.672	0.945
$MSDRP\_mu$	0.937	0.692	0.941
$MSDRP\_cnv$	0.903	0.681	0.943
$MSDRP\_CD$	0.898	0.664	0.947
$MSDRP$	**0.833**	**0.622**	**0.954**

References

[1] Yoon H, Ko Y H. LMP1+ SLAMF1high cells are associated with drug resistance in Epstein-Barr virus-positive Farage cells[J]. Oncotarget, 2017, 8(15): 24621.

[2] Jin S, Cojocari D, Purkal J J, et al. 5-Azacitidine induces NOXA to prime AML cells for venetoclax-mediated apoptosis[J]. Clinical Cancer Research, 2020, 26(13): 3371-3383.

[3] Edwards S K E, Han Y, Liu Y, et al. Signaling mechanisms of bortezomib in TRAF3-deficient mouse B lymphoma and human multiple myeloma cells[J]. Leukemia research, 2016, 41: 85-95.

[4] Mpakou V, Spathis A, Bouhla A, et al. Synergistic inhibitory effects of low-dose decitabine in combination with bortezomib in the AML cell line Kasumi-1[J]. Experimental and Therapeutic Medicine, 2021, 21(3): 1-1.

[5] Miedel M T, Graf N J, Stephen K E, et al. A pro-cathepsin L mutant is a luminal substrate for endoplasmic-reticulum-associated degradation in C. elegans[J]. PloS one, 2012, 7(7): e40145.

[6] Pemovska T, Bigenzahn J W, Srndic I, et al. Metabolic drug survey highlights cancer cell dependencies and vulnerabilities[J]. Nature Communications, 2021, 12(1): 7190.

[7] Abbou S, Lanvers-Kaminsky C, Daudigeos-Dubus E, et al. Polo-like kinase inhibitor volasertib exhibits antitumor activity and synergy with vincristine in pediatric malignancies[J]. Anti-cancer research, 2016, 36(2): 599-609.

[8] Yadav B, Pemovska T, Szwajda A, et al. Drug sensitivity scoring pipeline[J].

[9] Cher C Y, Man C H, Lam S S Y, et al. Targeting Polo-like Kinase in Acute Myeloid Leukemia[J]. 2014.

[10] Wang J, Beauchemin M, Bertrand R. Bcl-xL phosphorylation at Ser49 by polo kinase 3 during cell cycle progression and checkpoints[J]. Cellular signalling, 2011, 23(12): 2030-2038.

[11] Lehal R, Zaric J, Vigolo M, et al. Pharmacological disruption of the Notch transcription factor complex[J]. Proceedings of the National Academy of Sciences, 2020, 117(28): 16292-16301.

[12] Matsumoto S, Biniecka P, Bellotti A, et al. Nicotinaldehyde, a Novel Precursor of NAD Biosynthesis, Abrogates the Anti-Cancer Activity of an NAD-Lowering Agent in Leukemia[J]. Cancers, 2023, 15(3): 787.

[13] Kacsinta A D, Dowdy S F. Current views on inducing synthetic lethal RNAi responses in the treatment of cancer[J]. Expert Opinion on Biological Therapy, 2016, 16(2): 161-172.

[14] Ueda A, Oikawa K, Fujita K, et al. Therapeutic potential of PLK1 inhibition in triple-negative breast cancer[J]. Laboratory investigation, 2019, 99(9): 1275-1286.

[15] Oliveira J C, Pezuk J A, Brassesco M S, et al. PLK1 expression and BI 2536 effects in childhood acute lymphoblastic leukemia[J]. Pediatric Blood & Cancer, 2014, 61(7): 1227-1231.
